# Are single-use scopes the way of the future?

**DOI:** 10.1016/j.igie.2022.10.008

**Published:** 2022-11-04

**Authors:** Michael Jones, Linda S. Lee

**Affiliations:** 1 Division of Endoscopy, Boston Scientific, USA; 2 Division of Gastroenterology, Hepatology and Endoscopy, Brigham and Women’s Hospital, Boston, Massachusetts, USA

## Editor’s Foreword

Industry is a critical partner to promoting innovation in endoscopy. In this inaugural edition of *iGIE*, I am delighted to establish a section bringing the industry perspective to investigation and innovation in endoscopy. Although I have always been aware of the presence of industry in endoscopy, my true appreciation for their important role in fostering innovation within our field as well as training and investigation developed as I partnered more closely with them in my role as Medical Director of Endoscopy. I hope that giving voice to industry across various aspects of endoscopy will allow clinicians to appreciate their tremendous work in developing our field and to promote further investigation and innovation in endoscopy.

## Editor’s Introduction

**Figure 1:**
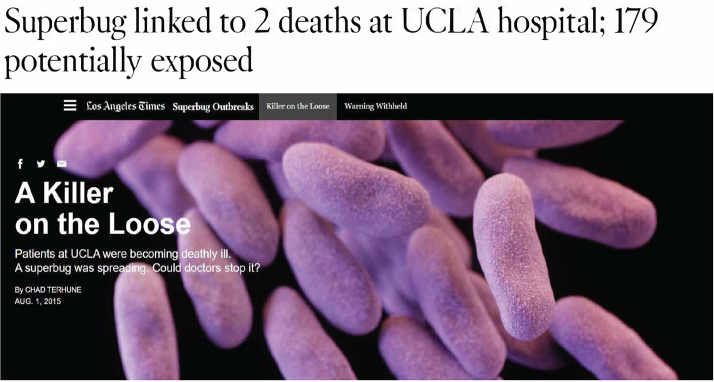
Los Angeles Times articles reporting on duodenoscope-related infections in 2015 (February 18, 2015 and August 1, 2015).

“Deadly infections from medical scopes go unreported, raising health concerns.” This was the headline from *USA Today* on August 5, 2015 as national media attention turned to our humble reusable duodenoscope, in use for the past several decades. Many other similar reports surfaced as hitherto unprecedented attention focused on the elevator and infection control processes, with culturing of scopes becoming the norm (Fig. 1). Although single-use scopes were certainly around even before all the media hype, these media-grabbing events were pivotal in propelling the marketing of single-use scopes.

Dating back to the early 1990s, concern over proper reprocessing of reusable flexible endoscopes led to investigations of disposable sheathed flexible sigmoidoscopes with the air–water, suction channels incorporated into the sheath and removable knobs from the now-defunct Vision Sciences. The scope-related patient infection rate was reported at 6%, although back then “high numbers of endoscopists were not convinced of the importance of reprocessing.” (p. 566)^1^ This randomized study noted decreased turnover time with reprocessing personnel favoring the disposable option, whereas endoscopists preferred the standard sigmoidoscope that more reliably reached 60 cm. This technology was adapted to the gastroscope, with another randomized trial in 1999 similarly noting shorter room turnover but a decreased ability to advance beyond the bulb with the sheathed scope.^2^ Not until this year in *Gastrointestinal Endoscopy* was a single-use gastroscope (Huizhou Xianzan Technology Co, Ltd, Huizhou, China) demonstrated to be noninferior to a standard reusable gastroscope for photodocumentation of standard locations, maneuverability, completion rate, and safety.^3^

Predictably, multiple companies including Ambu (Ballerup, Denmark), GI View Ltd (Ramat Gan, Israel), and ERA Endoscopy SRL (Cascina PI, Italy) are developing single-use colonoscopes, some combined with robotic capabilities. An interesting study exploring the economics of reusable colonoscopes using real-world data included costs to purchase, maintain, and reprocess these scopes as well as the cost of postprocedural infections.^4^ Per-procedure costs ranged from approximately $200 to $550, depending on the total number of colonoscopes available at an endoscopy center and total number of procedures performed annually. A similar analysis for disposable duodenoscopes noted the break-even cost at low-volume centers (≤50 ERCPs annually) was ≥$1300 compared with ≥$800 for high-volume centers (≥150 ERCPs annually) with cost per procedure ranging from $612 to $1362 for reusable duodenoscopes.^5^ Although economics is an important factor influencing an endoscopy center’s decision on using single-use scopes, noninferior (or superior) usability compared with reusable scopes remains one of the most important considerations.

Boston Scientific introduced the first U.S. Food and Drug Administration (FDA)-approved single-use duodenoscope in 2019. Therefore, it is fitting that our first conversation in the Industry Perspective section of *iGIE* is with Michael Jones, Senior Vice President and President, Endoscopy for Boston Scientific, a position he has held since May 2022. In this role he is responsible for the Endoscopy business, leading development and bringing to market devices to diagnose and treat a broad range of GI and pulmonary conditions, with innovative, less-invasive technologies. Michael earned a BA in Business from Colorado State University and has been with Boston Scientific for more than 25 years where he previously served as the Senior Vice President and General Manager, Endoscopy, before taking his current role. Michael also serves as the executive sponsor of the internal Boston Scientific VETS employee resource group, which is focused on cultivating gratitude and service to others, particularly active-duty veterans and their families.

**Figure 2:**
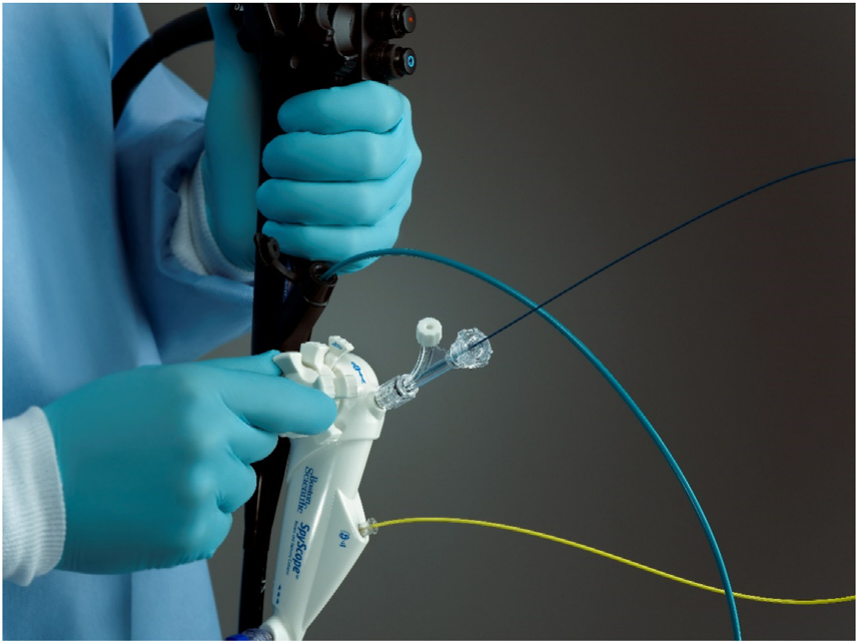
Original single-use, single-operator cholangioscope.

**Linda Lee (LL): Mike, thank you so much for taking time out of your incredibly busy schedule to discuss single-use scopes. Would you discuss the history of single-use scopes? (** Fig. 2 **)**

**Michael Jones (MJ):** The innovation toward single-use endoscopes has been rooted in the need to address unmet clinical needs. To understand those needs, it’s worth looking back to the late 1960s when endoscopists first began performing ERCP. One challenge endoscopists experienced was the direct examination of the biliary tree. It wasn’t until the 1980s when mother–daughter systems were introduced as a solution to this.^6^

These systems were comprised of a large-channel duodenoscope (“the mother”) through which a smaller cholangiopancreatoscope (“the daughter”) could be inserted. By design, mother–daughter systems required 2 endoscopists. These reusable systems were also fiberoptic, and the bundles were fragile and prone to breaking.^6^ Cholangioscopy performed using this system was cumbersome, labor intensive, and time-consuming.^7^ The combination of these matters led to insufficiencies and potentially resulted in less-than-optimal outcomes.

In 2007, we launched the first single-use, single-operator cholangioscope system worldwide, revolutionizing the endoscopy field. This system enables direct visualization and guides accessory devices for diagnostic and therapeutic procedures in the pancreatic and biliary system. We continued to innovate this system and in 2015 received FDA clearance on our next generation, which further improved visualization and functionality.

**LL: Why has the momentum in the shift toward single-use scopes been slower than anticipated?**

**MJ:** With any new technology it takes time for adoption to happen, and change does not occur overnight. This was true with single-operator cholangioscopy, yet now it is has gained widespread acceptance as the standard technique.^7^ When we approached our more recent single-use scopes, we knew the task we were facing and the amount of education needed to ensure physicians’ success when adopting future single-use scopes.

The first fully single-use duodenoscope was granted FDA 510(k) clearance in December of 2019 and was developed as an alternative to reusable duodenoscopes to address the risk of patient-to-patient infection and eliminate the need for reprocessing and repairs. It allowed physicians to use a new, sterile device for every procedure and was built on the familiar design of reusable duodenoscopes so that physicians could experience a minimal learning curve when adopting the new technology.

As we launched the single-use duodenoscope, we understood that before physicians could adopt this technology, they needed to understand the benefits it would bring to their patients and staff. They also needed to trust that the device would operate as they needed it to. This type of behavior change took time and a lot of education, especially when physicians were already operating reusable duodenoscopes that had worked well for them for years.

**Figure 3:**
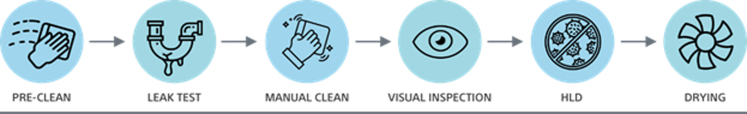
From precleaning and leak testing to HLD and drying, there can be more than 100 distinct steps to reprocessing a reusable duodenoscope, all of which introduce the opportunity for patient cross-contamination.

**LL: Why is there all this interest in this technology now? (** Fig. 3 **)**

**MJ:** Interest in single-use scopes has been building for multiple years since their commercialization. Education has been key to building momentum around these new technologies. Beyond preventing scope-related, patient-to-patient infections, single-use scopes may help improve operational efficiencies, which has become a key driver in adoption of these technologies. We’re aware that device reprocessing for reusable scopes ties up staff for extended periods of time and requires a significant financial and time-intensive investment for training and credentialing.

Because of the COVID-19 [coronavirus disease 2019] pandemic, hospitals are experiencing a strain on resources. Some single-use scopes can reduce operational burdens for hospitals by removing any unplanned downtime because of equipment out for repair or having to wait for the scopes to be cleaned. For example, reusable scopes may require maintenance, which requires scheduling with an outside party in order for the scope to be repaired. Hospitals have a set number of scopes, and when these scopes are undergoing maintenance or reprocessing, they are unavailable for extended periods of time so hospitals can only complete a finite number of procedures. Procedures may be delayed or spaced further apart as a result.

Coupled with this was the FDA issuing communications around the risk of infection associated with various types of reusable and reprocessed endoscopes (duodenoscopes, bronchoscopes, urologic scopes). Specific to duodenoscopes, the FDA recommended providers transition to partially or fully single-use duodenoscopes in 2019.^8^ In April 2022, the FDA strengthened guidance recommending hospitals complete the transition to innovative and single-use duodenoscopes as a result of data showing that up to 6% of reusable duodenoscopes tested positive for “high-concern organisms” associated with disease.^9^ The FDA’s focus on this issue has added weight and priority to the conversation around scope-related infections.

**Figure 4:**
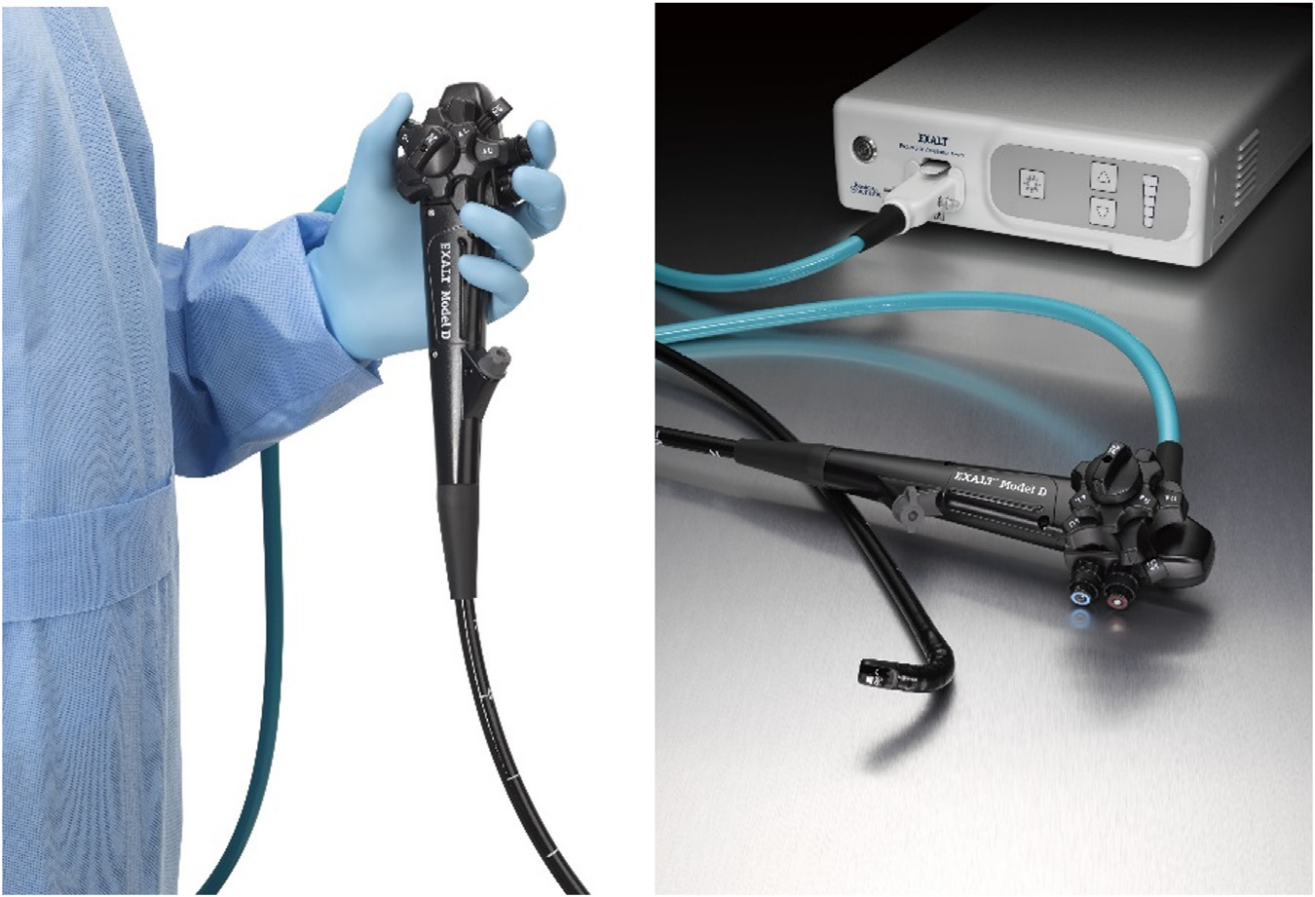
First-generation single-use duodenoscope. The first single-use duodenoscope on the market was granted Breakthrough Device Designation and cleared by the U.S. Food and Drug Administration in 2019.

**Figure 5:**
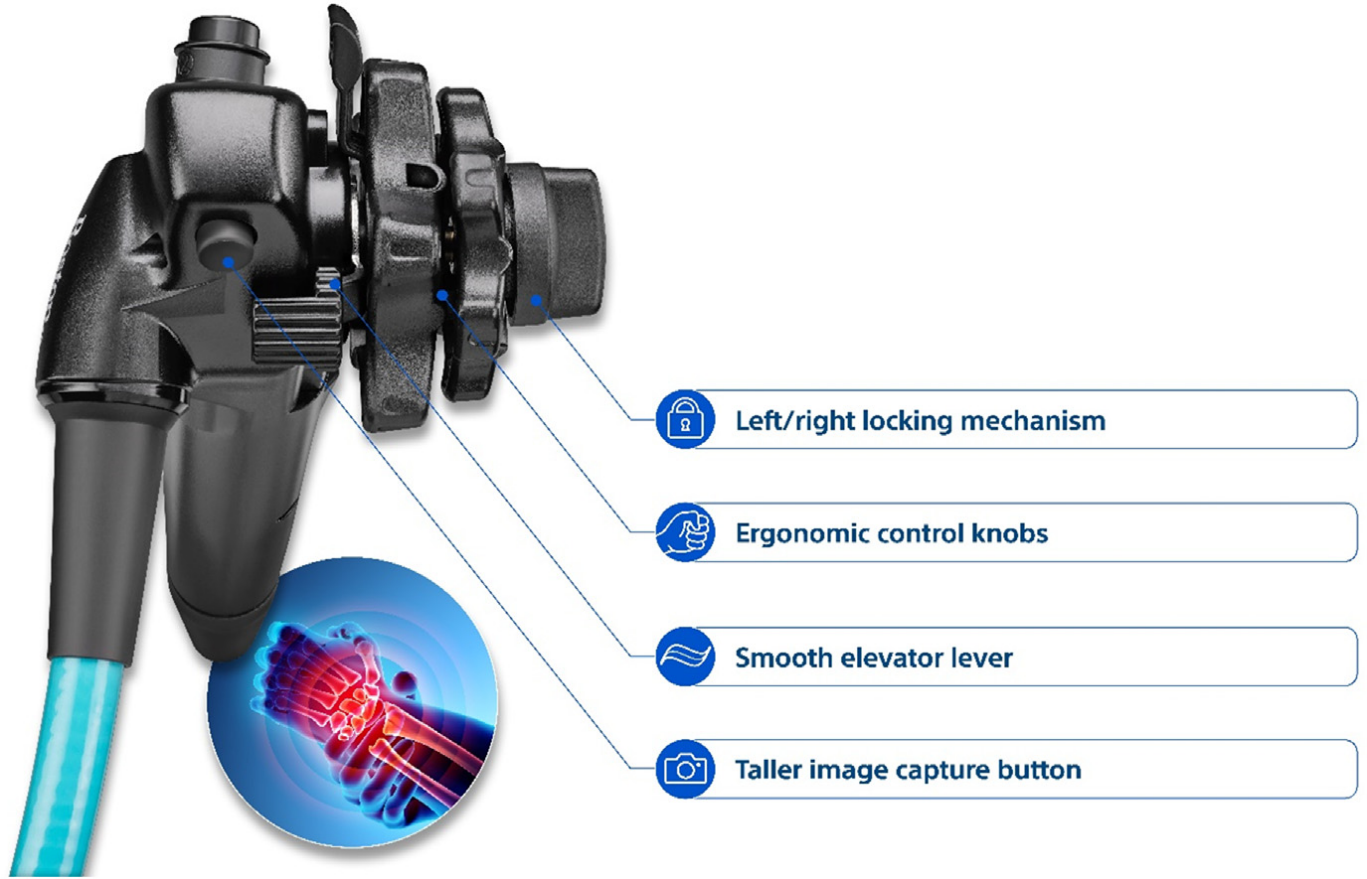
Latest version of the single-use duodenoscope. Driven by direct feedback from gastroenterologists, rapid updates to the first single-use duodenoscope have helped make it more ergonomic.

**LL: What challenges have there been and continue to be in developing this technology? (** Figure 4, Figure 5 **)**

**MJ:** As we started developing single-use scopes, we knew we would need to deliver comparable performance with a reusable scope. This was critical to adoption and one of our main priorities during development because the physician would need to get comfortable with how a single-use scope feels and potentially would need to adjust their technique.

Duodenoscopes are incredibly complex to manufacture. When it comes to functionality, duodenoscopes must be flexible enough to navigate a tortuous GI tract but stiff enough to effectively deliver devices to a patient’s biliary anatomy. Single-use scopes must handle similarly and must also effectively meet clinician expectations. Making duodenoscopes in a single-use version was a huge engineering feat because of these factors. In fact, the endoscopy team behind research and development is comprised of over 100 people, all working toward the goal of innovation. Since launch, we’ve continued to improve our single-use duodenoscope by making ergonomic enhancements to meet the familiar feel physicians are used to with a reusable scope.

As we look to future innovation, we are pushing ourselves to drive clinical differentiation. When it comes to developing any new technology, our R&D [research and development] team works closely with physicians to identify unmet healthcare needs; for example, exploring ERCP solutions to improve outcomes for patients with challenging or altered anatomies. Our team focuses innovation based on this input and re-engages clinicians at multiple touchpoints along the way to ensure our designs are meeting clinician expectations. An advantage to single-use devices is that they can be iterated quickly based on physician feedback, and the next, improved version of the device can get into physicians’ hands earlier, whereas with reusable devices, a customer will purchase it once and continue to use it for quite some time before they replace it with the next version.

Making a single-use duodenoscope was a significant technologic accomplishment and with that comes great responsibility. We are committed to patient safety and demonstrating to our physicians that it can stand with the performance of a reusable scope.

**Figure 6:**
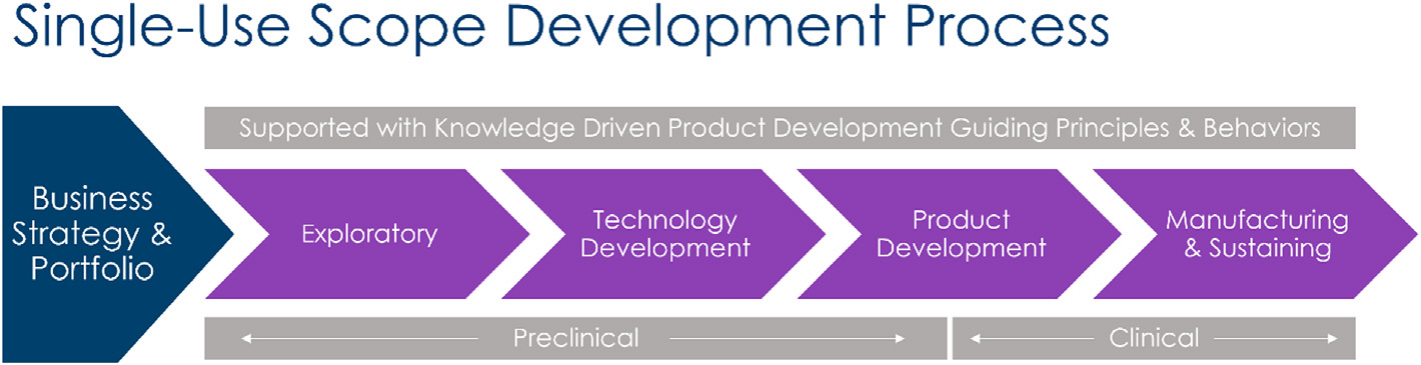
Flowchart of company’s process in bringing to market clinically used, single-use scope. This scalable process integrates business, technical, and quality system tasks to drive innovative product ideas from strategy through commercialization.

**LL: What steps have been critical to bringing a single-use scope to market? (** Fig. 6 **)**

**MJ:** The needs of physicians and their patients were at the front of our minds as we introduced our single-use devices to the market.

One of the biggest challenges hospitals are facing is staff capacity. Increasing efficiencies at care sites is critically important to ensuring physicians are equipped to treat patients in a timely manner. One of the major benefits physicians helped us to identify, as we brought our single-use devices to market, was the ability to help alleviate operation and staff burdens. These devices are ready when needed, no repairs or maintenance are needed, and there is no unplanned downtime because of waiting for scopes to be cleaned. Staff are not tied up cleaning these devices. This is a huge win for care staff, particularly for procedures that are scheduled on short turnaround or during times when there is reduced staff available on site to support device reprocessing.

Critical to adoption was the preservation of quality and functionality that physicians experienced with their reusable scopes. For our single-use duodenoscope, for example, we designed our device to feel intuitive to physicians who were used to a reusable scope. To demonstrate usability, we conducted 2 voluntary, prospective clinical trials that were published in peer-reviewed journals.^10^^,^^11^ Endoscopists used the device to complete ERCPs across cases of varying complexity. The findings have helped to demonstrate a high procedure success rate and physician satisfaction with a single-use duodenoscope, which helped to support physician confidence in a new single-use device. In addition, 4 other independent studies have been published to reinforce these findings.^12^, ^13^, ^14^, ^15^

We also supported physicians with a peer-to-peer educational program to help endoscopists understand the nuances of using a single-use duodenoscope. This helped physicians become comfortable with the new technology so they could hone their skills and adjust their technique as needed.

It was also critical to ensure that it was financially viable for hospitals to introduce single-use duodenoscopes into their systems. To support this, our Health Economics and Market Access team applied for and secured temporary Medicare device-specific payments known as New Technology Add-On Payment for inpatient procedures and Transitional Pass-Through payment for outpatient procedures. The intent of these payments is to foster innovation and increase Medicare beneficiary access to new technologies that improve clinical care. The approval of these device-specific payments has supported patient access to single-use duodenoscopes.

**LL: How do you answer the criticism that creating all these plastic single-use scopes is not environmentally friendly?**

**MJ:** As manufacturers, we are committed to caring for our environment, including identifying areas to reduce waste, off-set environmental impact, and enable our customers to responsibly recycle our single-use devices. There is limited research available on the environmental impact of single-use scopes compared with reusable scopes. A study comparing the carbon footprint of a single-use versus a reusable ureteroscope found that they were comparable.^16^ Evaluating the environmental impact of both single-use and reusable devices remains complex and may include some less-obvious factors. For example, because single-use scopes eliminate the need for reprocessing, they also reduce the amount of chemicals, materials, and personal protective equipment dedicated to reprocessing.

To help off-set the environmental impact, we offer a no-cost recycling program for our single-use duodenoscopes to many hospitals across the nation and encourage our customers to take advantage of this program. We also continuously evaluate other ways to reduce waste in the design of future products by exploring materials and packaging options that leave a lesser environmental impact. Simultaneously, we are continuously considering options to off-set our environmental impact.

This is not only our approach when considering our single-use devices, but it is a philosophy that is important to us as a company, and we believe demonstrates a commitment to the sustainability of our planet. We are proud to lead by example as one of the first medical device manufacturers to establish a goal to achieve carbon neutrality by 2030.

As part of this conversation, we also need to consider what’s best for the patient. Single-use scopes were developed to eliminate scope-related, patient-to-patient infections, which may also lead to an additional hospital stay, antibiotics, and care. We strive to always do what is right for the patient and that means putting their safety and well-being first.

**LL: Would you discuss the role of single-use scopes in non-GI fields such as pulmonary, ear-nose-throat, urology, etc?**

**MJ:** Similar to duodenoscopes, the FDA has issued communications on bronchoscopes and urologic scopes. New guidance from the FDA on bronchoscope reprocessing and use recommended considering a single-use bronchoscope in situations of increased risk of spreading infection or when there is no immediate support for reprocessing of the bronchoscope. The FDA’s notice points to the American Association for Bronchology & Interventional Pulmonology’s recommendation to use single-use bronchoscopes as a first line for patients with suspected or confirmed COVID-19. Last year, the FDA issued a letter to healthcare providers noting the risk of infections associated with reprocessed urologic endoscopes, including cystoscopes, ureteroscopes, and cystourethroscopes.

Our product development is focused on the patient and the procedure. As devices are successful in solving clinical challenges in one area, we look at how these technologies can be used in other procedures. We are working to give more physicians access to technologies so more patients can be treated with less-invasive procedures, using less-invasive devices. For example, we leveraged our direct visualization platform to develop a digital visualization catheter to expand treatment options for surgeons and interventional radiologists managing biliary obstructive diseases. Our R&D team has helped address the issue of cross-contamination because of ineffective reprocessing∗ with a portfolio of single-use scopes used in bronchoscopy, urologic, and GI tract procedures.

**LL: What do you see as the future for single-use scopes in the United States and globally?**

**MJ:** Because of the operational flexibility and financial viability that single-use scopes offer hospitals, we believe the use of these devices will only continue to grow. For many physicians who have made the switch, there is less incentive to go back.

For example, our single-use duodenoscope is used to facilitate examination of the pancreatic and bile ducts—a complex and infection-sensitive procedure—while eliminating the possibility of an endoscope-acquired infection from reprocessing deficiencies. Our single-use cholangioscope allows physicians to directly visualize the interior of the pancreatic and bile ducts and concurrently treat stones or blockages.

As we look to the future of single-use scopes, unmet patient and clinical needs will continue to be a priority. By examining how to improve efficiencies, enhance clinical outcomes, and drive patient care, the innovations in single-use scopes will create new standards of care. Possibilities in procedural evolution and differentiated technologies include artificial intelligence and tissue characterization, data analytics for workflow optimization, and more.

Across many medical fields, outside of endoscopy, single-use devices are the standard of care. It only makes sense that the industry (meaning manufacturers, payers, and healthcare providers) moves in this direction. By putting the patient first, we are continuing to invest in the development of additional single-use devices for endoscopy. As we innovate our current products, we are committed to addressing physician and clinical needs to bring efficiencies that can be directly applied to patients.

## Editor’s Closing Remarks

A combination of national and international media attention on multidrug-resistant organism infections related to reusable duodenoscopes, FDA policy, 3-dimensional printing technology, and a company with experience in bringing a single-use cholangioscope to market all helped propel the first single-use duodenoscope to clinical use, which was no small technological feat, by Boston Scientific. Single-use scopes are attractive clinically in higher-risk patients including immunocompromised and COVID-19 patients, postsurgical anatomy if special single-use duodenoscopes can be designed to perform ERCPs, emergency after hours procedures, and hybrid procedures in the operating room requiring sterile scopes. Single-use scope technology can innovate rapidly, eliminate economic and environmental issues related to reprocessing, and improve efficiency in an endoscopy center. The overall economics of routinely using these scopes varies depending on each endoscopy center with its unique patient mix and reusable-scope associated infection rate and must include assessment of cost of scopes compared with potential savings from reprocessing and scope maintenance and repairs. Adoption of this technology more broadly is predicated on a variety of complex factors including the endoscopist, patient, and endoscopy center. Market penetration of single-use scopes remains unpredictable with further innovation required; however, this technology is clearly here to stay with an exciting future ahead.

## Disclosure

*The following authors disclosed financial relationships: M. Jones: Employee of Boston Scientific. L. S. Lee: Consultant for Fractyl, Boston Scientific, and Fujifilm Medical; research support from Fujifilm Medical.*

## Footnotes

∗ As defined by the American Society for Gastrointestinal Endoscopy.
